# Supplementary figures and images for: Exploiting bacterial effector proteins to uncover evolutionarily conserved antiviral host machinery
Source: PLoS Pathog. 2024 May 16;20(5):e1012010. doi: 10.1371/journal.ppat.1012010 (PMC11098378; doi:10.1371/journal.ppat.1012010)

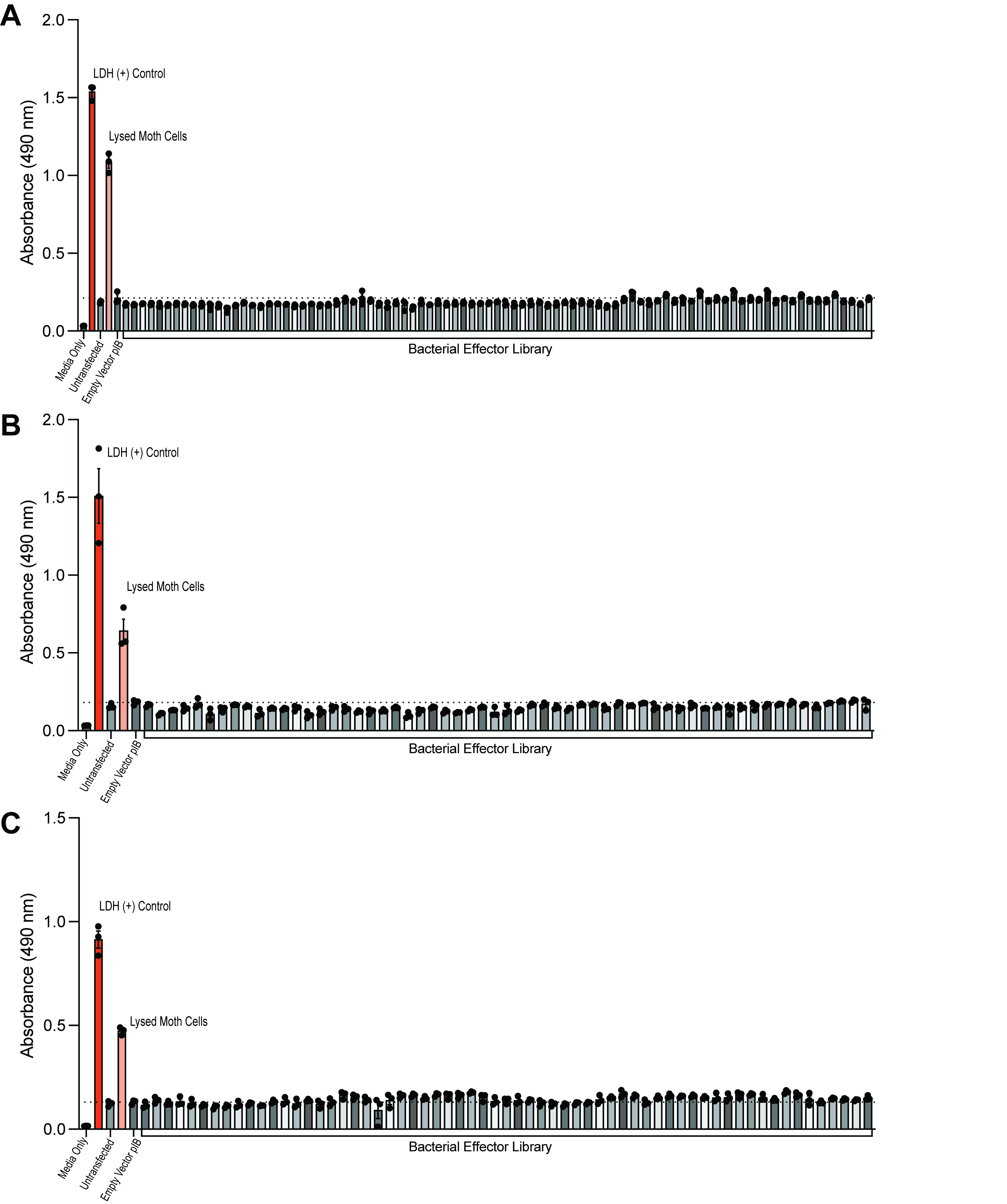

Supplement: S1 Fig — A-C. Absorbance at 490 nm for supernatant collected from LD652 cells expressing the pIB/V5-His-based effector library for 48 h. Source data for cell viability assays are available in S1 Table. (TIF) [file ppat.1012010.s006.tif]

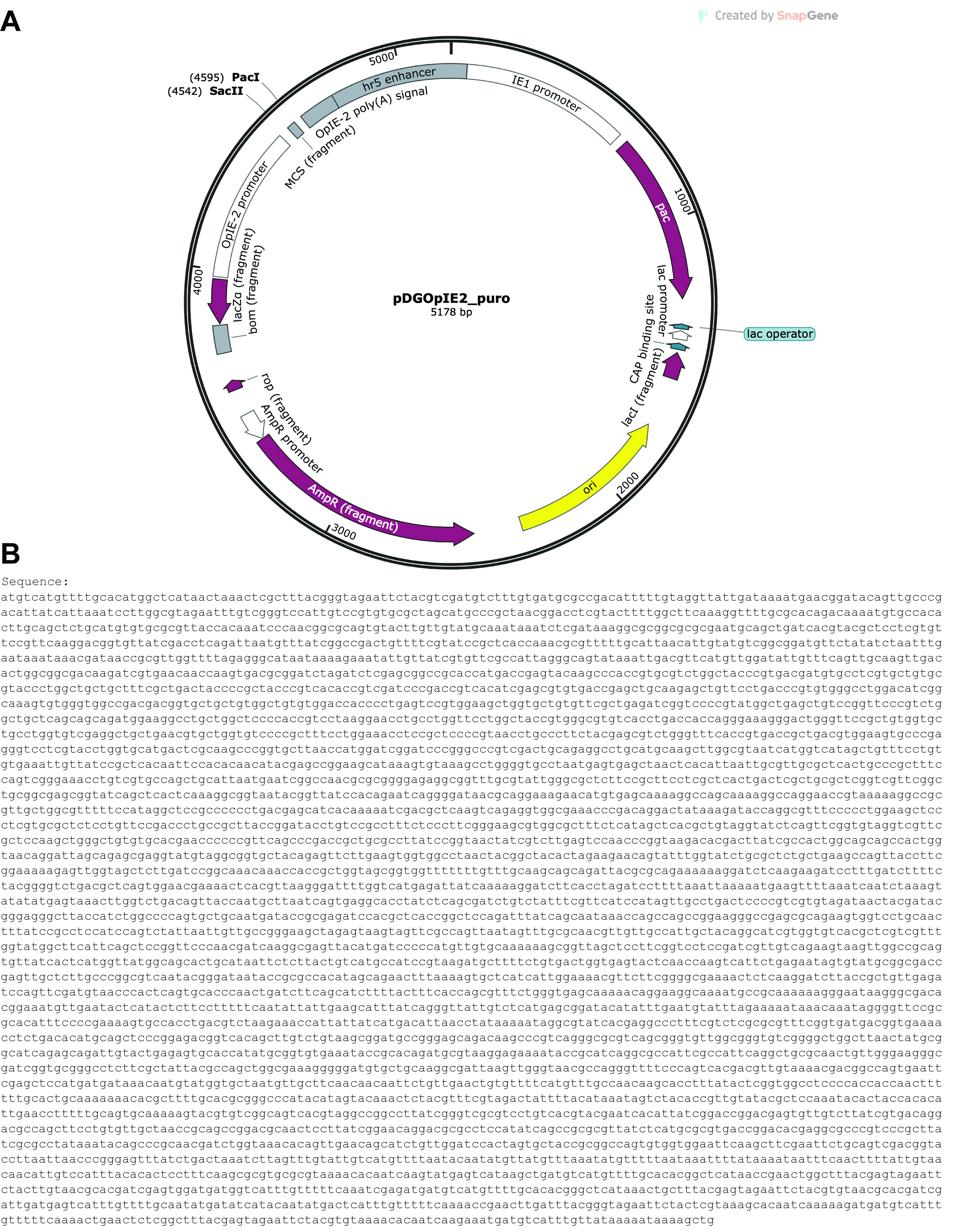

Supplement: S2 Fig — A. Snapgene vector map of pDGOpIE2 vector and features of interest. B. Complete sequence of pDGOpIE2 vector. (TIF) [file ppat.1012010.s007.tif]

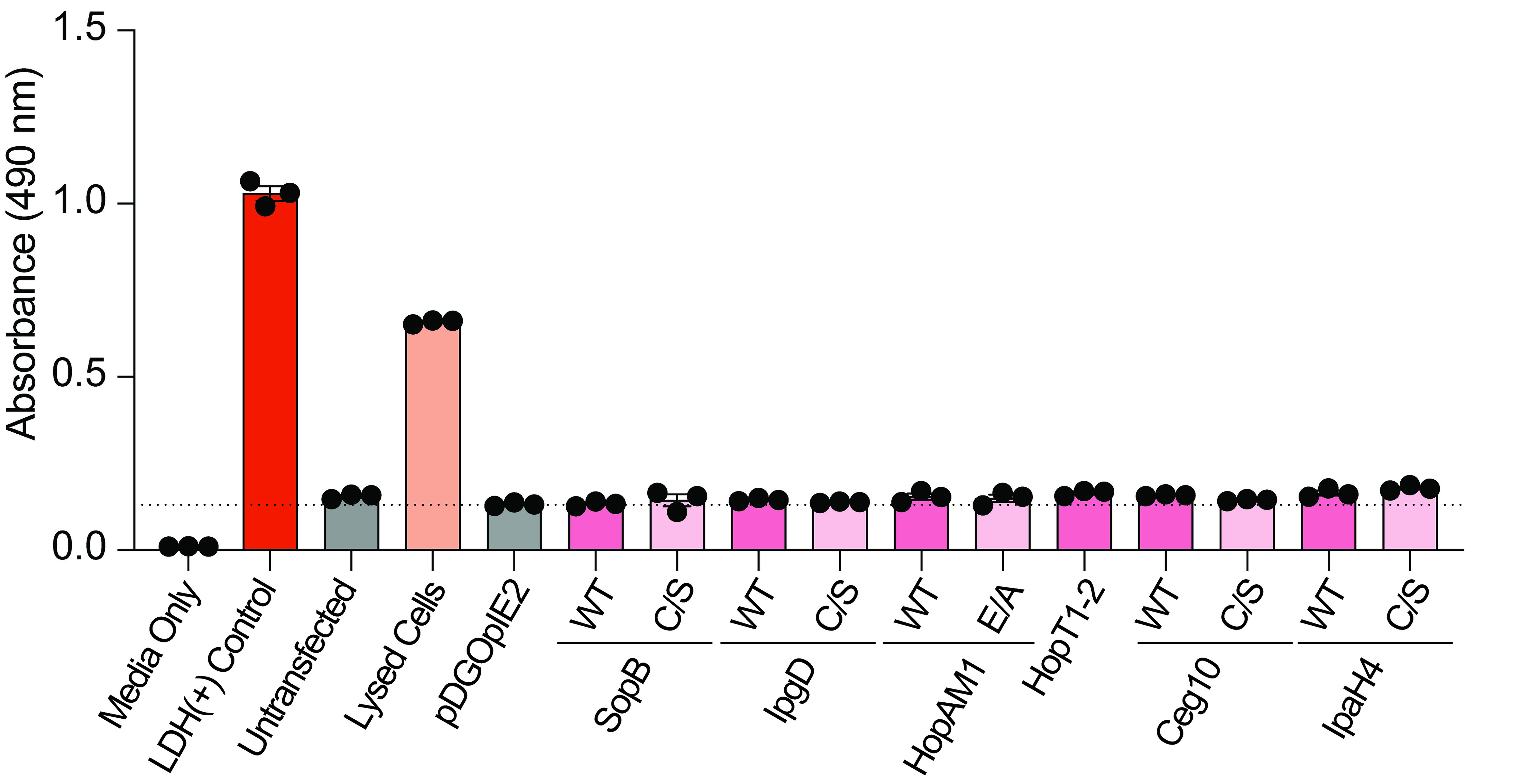

Supplement: S3 Fig — Absorbance at 490 nm for supernatants collected from LD652 cells transfected with the indicated pDGOpIE2 effector expression vectors for 48 h. (TIF) [file ppat.1012010.s008.tif]

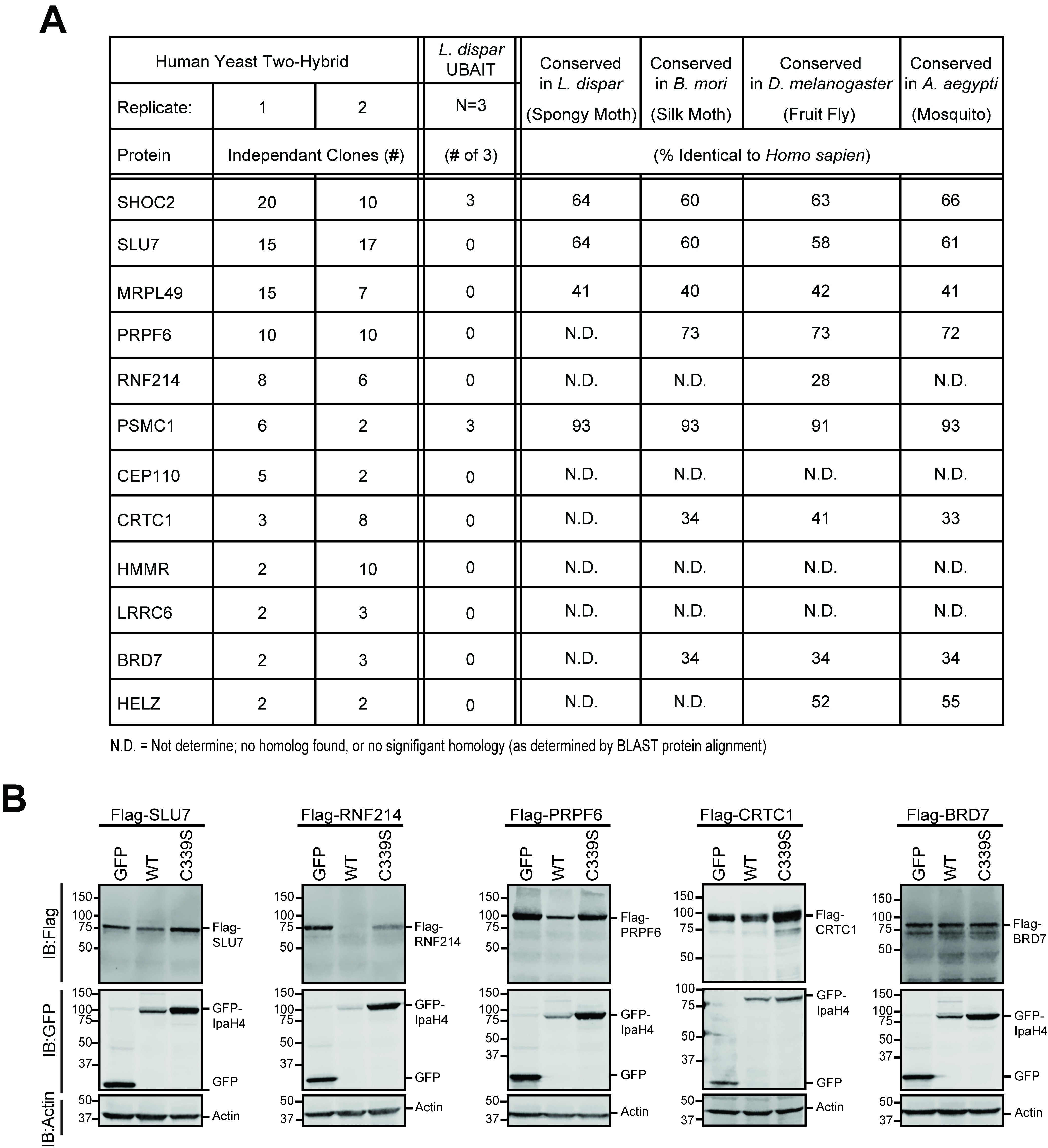

Supplement: S4 Fig — A. Table summarizing results of two independent Y2H screens using a human prey library and three independent ubiquitin-activated interaction trap (UBAIT) assays using LD652 cell lysate. Hits were then analyzed via Blastp to determine percent identity to their Homo sapiens ortholog. N.D. = Not determined; either no ortholog found or no significant homology (as determined by BLAST). (B) Representative immunoblot of degradation assays for Flag-tagged human proteins following 48 h co-expression in HEK293T cells with GFP, IpaH4 (WT) or catalytic mutant GST-IpaH4C339S (C339S). (TIF) [file ppat.1012010.s009.tif]

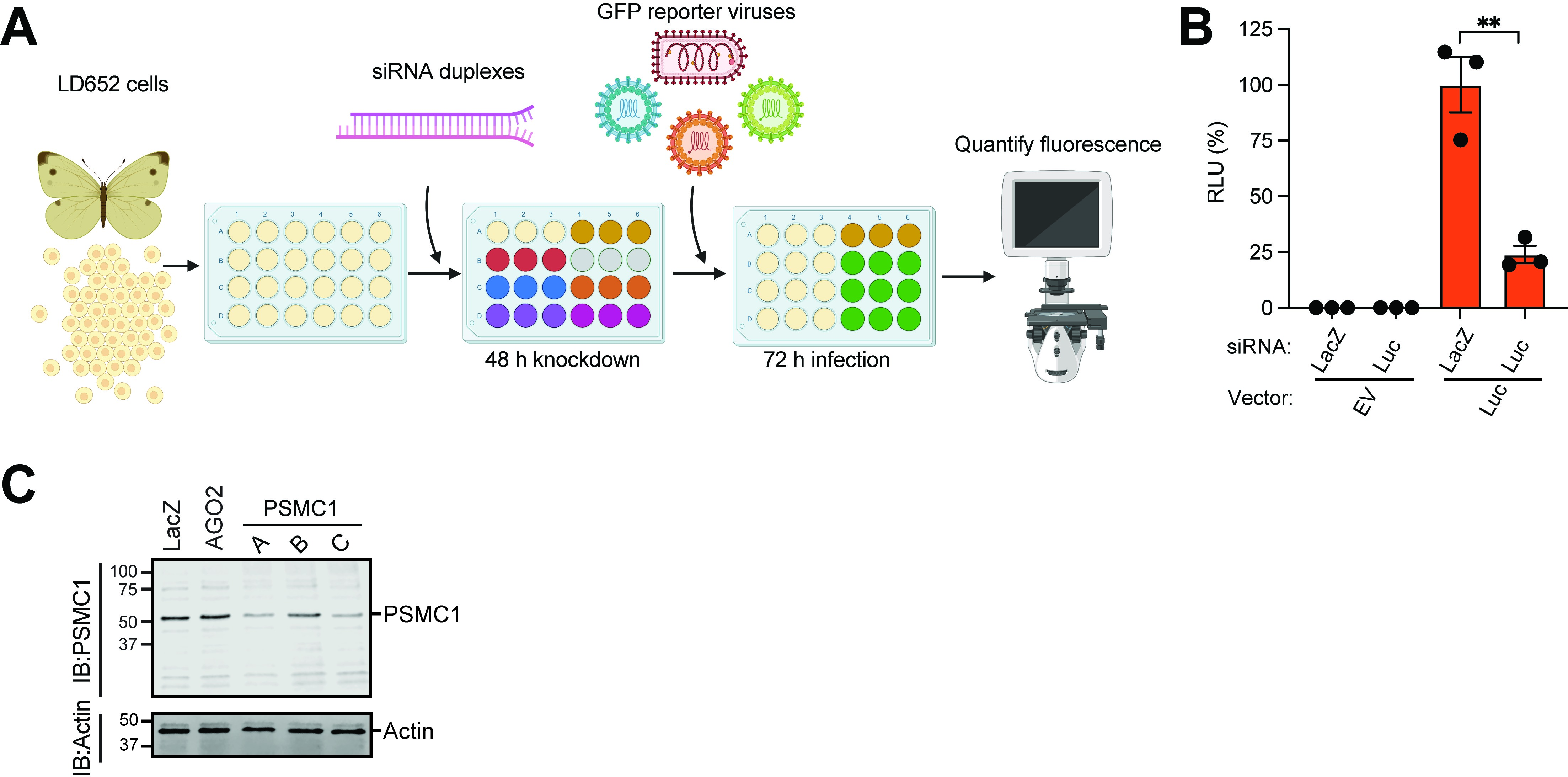

Supplement: S5 Fig — A. Schematic detailing siRNA knockdown protocol in LD652 cells. Image was created with BioRender.com. B. Relative Light Units (RLU) of LD652 lysates from cells transfected with empty vector (EV) or luciferase (Luc)-expressing vectors for 48 h and then transfected with siRNA targeting LacZ (negative control) or Luc sequences for 48 h. C. Representative immunoblot of PSMC1 knockdown following 48 h for siRNA treatment with duplexes targeting LacZ, AGO-2, or PSMC1. (TIF) [file ppat.1012010.s010.tif]

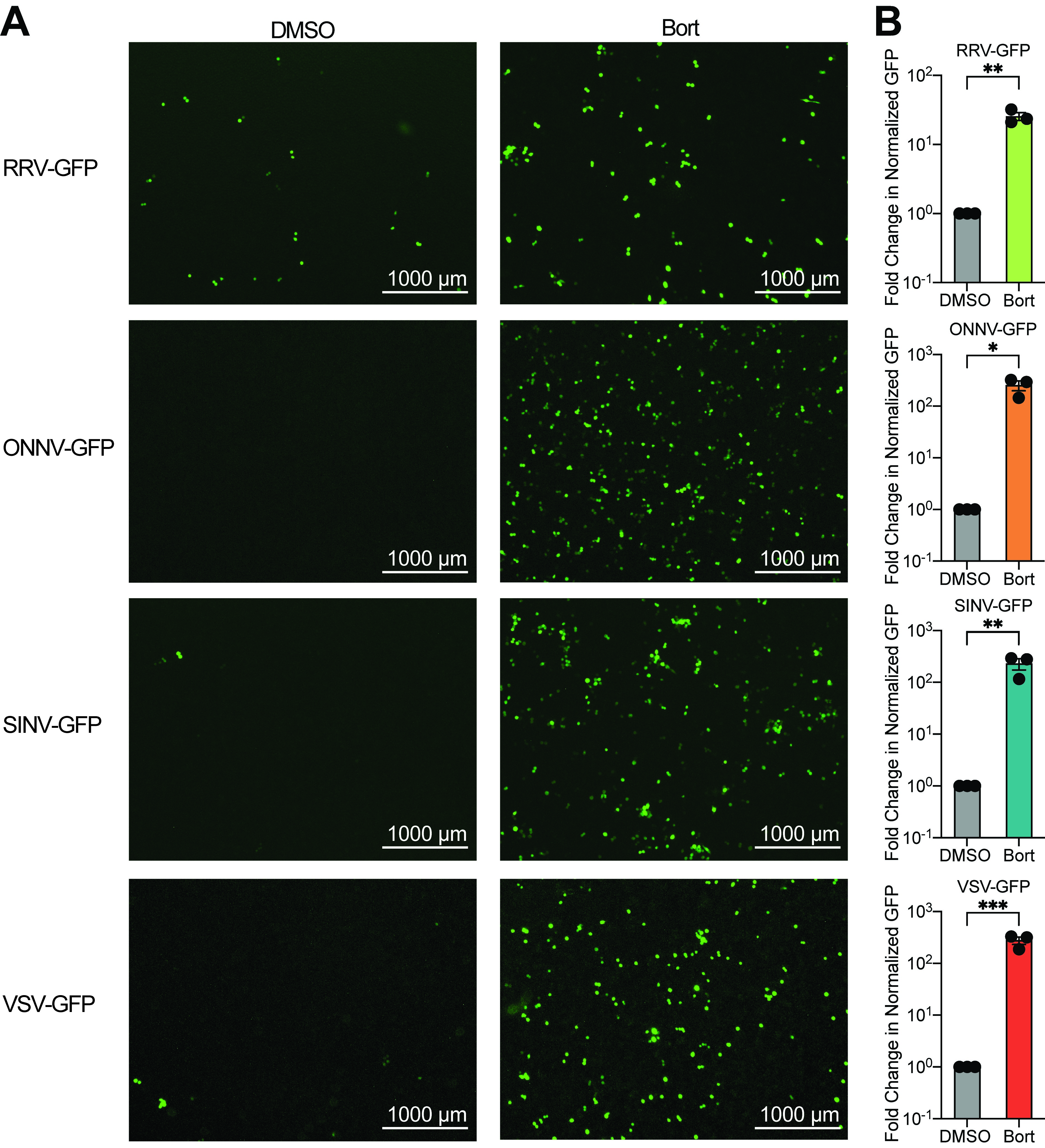

Supplement: S6 Fig — A. Representative fluorescence microscopy images (GFP channel) of LD652 cells 72 hpi with the indicated GFP reporter strains that were treated with DMSO (vehicle) or 50 nM Bortezomib (Bort). DMSO or Bort was added 2 hpi. B. Fold-change in normalized GFP signals in Bort-treated cultures relative to DMSO treatments. Cells were stained 72 hpi with CellTracker Orange dye (not shown) and imaged in GFP and RFP channels to calculate fold-change in GFP signal after normalization of cell number using CellTracker (RFP) channel signals. Data are means ± SD; n = 3. Statistical significance was determined with unpaired student’s t-test; ns = P>0.1234, * = P<0.0332, ** = P<0.0021, *** = P<0.0002, **** = P<0.0001. (TIF) [file ppat.1012010.s011.tif]
